# Inhibition of MAVS Aggregation-Mediated Type-I Interferon Signaling by Foot-and-Mouth Disease Virus VP3

**DOI:** 10.3390/v13091776

**Published:** 2021-09-06

**Authors:** Pathum Ekanayaka, Byeong-Hoon Lee, Asela Weerawardhana, Kiramage Chathuranga, Jong-Hyeon Park, Jong-Soo Lee

**Affiliations:** 1 College of Veterinary Medicine, Chungnam National University, 220 Gung-dong, Yuseong-gu, Daejeon 34134, Korea; ekanayaka16@gmail.com (P.E.); byeonghoon_2@naver.com (B.-H.L.); aselasampath2009@gmail.com (A.W.); chathurangakiramage@gmail.com (K.C.); 2 Animal and Plant Quarantine Agency, 177 Hyeoksin 8-ro, Gyeongsangbuk-do, Gimcheon-si 39660, Korea; parkjhvet@korea.kr

**Keywords:** FMDV VP3, MAVS, mitochondria localization, aggregation, interferon

## Abstract

As a structural protein of the Foot-and-mouth disease virus (FMDV), VP3 plays a vital role in virus assembly and inhibiting the interferon (IFN) signal transduction to promote FMDV replication. Previous studies demonstrated that FMDV VP3 blocks the type-I IFN response by inhibiting the mRNA expression of the mitochondrial antiviral-signaling protein (MAVS); however, the underlying mechanism is poorly understood. Here, we describe the specificity of FMDV VP3 interaction with the transmembrane (TM) domain of MAVS as FMDV driven type-I IFN inhibitory mechanism for its effective replication. The TM domain of MAVS governs the mitochondria localization of MAVS, and it is a key factor in type-I IFN signaling transduction via MAVS aggregation. Thereby, the interaction of FMDV VP3 with the TM domain of MAVS leads to the inhibition of MAVS mitochondria localization, self-association, and aggregation, resulting in the suppression of type-I IFN response. Collectively, these results provide a clear understanding of a key molecular mechanism used by the FMDV VP3 for the suppression of IFN responses via targeting MAVS.

## 1. Introduction

Foot-and-mouth disease (FMD) virus is a highly contagious and economically devastating virus that infects wild and domestic cloven-hoofed animals and poses a major threat to the livestock industry worldwide [1,2,3]. The foot-and-mouth disease virus (FMDV), the etiological agent of FMD, is a positive-sense single-stranded RNA virus [4,5] of genus Aphthovirus within family *Picornaviridae* [6] and has seven immunologically distinct serotypes (O, A, C, Asia1, SAT1, SAT2, and SAT3) [2,7]. The ~8500 nucleotide genome is translated into a single polyprotein [7,8] that is post-translationally processed into four structural (VP1, VP2, VP3, and VP4) and ten non-structural proteins (Lpro, 2A, 2B, 2C, 3A, 3B1-3, 3Cpro, and 3D) with the help of virus-encoded proteases [7,8].

Virally infected host cells produce IFNs, which are essential components of the innate immune response [9,10,11] following the activation of cell-surface or intracellular pattern recognition receptors (PRRs) [12]. The type-I IFNs, including IFN-α and IFN-β, are produced by almost all cell types and play a vital role in host defense against viral infections [9,10,11]. The PRRs, mainly retinoic acid-inducible gene I (RIG-I) and melanoma differentiation-associated gene 5 (MDA5), detect initial virus infection by recognizing viral RNA in the cytoplasm of the cells [13]. Both RIG-I and MDA5 have two caspase recruitment domains (CARDs) at the N terminus; after activation of the PRR by viral infection, the CARDs of RIG-I or MDA5 interact with the CARD of mitochondrial antiviral signaling protein (MAVS; also known as IPS-1, VISA, and Cardif), providing an initial signal for MAVS activation [14,15,16,17]. MAVS is mainly localized in the mitochondria of the cells. Besides localizing at mitochondria, MAVS does also localize at peroxisomes and mitochondria-associated membranes (MAM) [18,19]. Furthermore, membrane localization of MAVS allows its aggregation. In particular, mitochondrial localization of MAVS is necessary for proper activation of the protein; once activated, MAVS aggregates by forming a functional prion-like structure on mitochondria [20]. This structure serves as a platform to create the MAVS signalosome, which primes the activation of TBK1 and IKK-ε [20,21,22], which locates downstream to the MAVS in the type-I IFN pathway. This leads, in turn, to the activation of downstream molecules IRF3, IRF7, and NF-κB (activated via IKK) of the type-I IFN signaling cascade, which elicits antiviral responses through the production of IFNs [14,15,16,17].

To propagate rapidly and efficiently at the initial site of infection, FMDV has evolved multiple immune evasion mechanisms to counteract type-I IFN responses [23,24,25]. Several FMDV proteins participate in host immune evasion. The FMDV proteases, Lpro and 3Cpro, function in the IFN-related signal transduction pathway and inhibit the host translation system [26,27]. Importantly, FMDV Lpro functions as a viral deubiquitinase, targeting RIG-I, TBK1, TRAF3, and TRAF6 for deubiquitination and thereby suppressing the type-I IFN signaling cascade [28]. Additionally, Lpro cleaves the p65 subunit of NF-ĸB [29,30], thereby suppressing the IRF3/7 expression [31,32]. FMDV 3Cpro cleaves NEMO [33] and inhibits nuclear translocation of STAT1 [34]. In addition, 3Cpro degrades RIG-I, MDA5 [33,35], and LGP2 [36], which are PRRs of the type-I IFN pathway that recognize viral RNA. It also degrades the proteins of the ATG5–ATG12 complex, which is involved in autophagy and NF-ĸB antiviral responses [37], and degrades PKR to facilitate viral replication [38]. FMDV 2B interacts with RIG-I and LGP2 to impair antiviral signal transduction [36,39]. FMDV 3A is responsible for the DDX56-mediated inhibition of IRF3 phosphorylation [40] and the transcriptional inhibition of RIG-I, MDA5, and MAVS [41]. Further, FMDV 3A promotes the upregulation of LRRC25-mediated G3BP1 degradation, thereby inhibiting RIG-I and MDA5 expression [42]. Concerning the FMDV structural proteins, the function of MAVS interaction and subsequent MAVS/TRAF3 interaction inhibition was identified in relation to FMDV VP1 [43]. FMDV VP1 interacts with sorcin to inhibit the type-I IFN cascade [44,45]. FMDV VP3 inhibits expression of MAVS protein by disrupting its mRNA, thereby contributing to the evasion of type-I IFN responses [46]; however, the underlying mechanism is unclear.

In this study, we further characterized the involvement of FMDV VP3 in suppressing the type-I IFN response. Our findings reveal the precise molecular mechanism by which FMDV VP3 contributes to the evasion of antiviral responses by targeting MAVS in the type-I IFN pathway.

## 2. Materials and Methods

### 2.1. Cells and Viruses

Porcine kidney (PK15), mouse leukemic monocyte macrophage (Raw264.7), human embryonic kidney 293 (HEK293T), and HeLa cell lines were cultured in Dulbecco’s modified Eagle’s medium (DMEM) (Gibco, Thermo Fisher Scientific, Waltham, MA, USA). All the media were supplemented with 10% fetal bovine serum (FBS) (Gibco) and 1% antibiotic/antimycotic (Thermo Fisher Scientific, Waltham, MA, USA). Cells were incubated in a humidified incubator at 5% CO_2_ and 37 °C atmospheres. Influenza virus A H1N1 strain A/PR/8/34, Sendai virus Cantell strain, GFP tagged PR8 (PR8-GFP) virus, and GFP tagged VSV (VSV-GFP) virus were used for our experiments. The influenza virus A H1N1 and Sendai virus was amplified in the specific-pathogen-free embryonated chicken eggs and the VSV-GFP virus was amplified in Vero cells. For the generation of the PR8-GFP virus, the A/PR/8/34 strain of influenza A virus (IAV) was used. In brief, for the generation of IAV carrying a fluorescent marker, the green fluorescent protein (GFP) gene was fused to the gene of the nonstructural protein 1 (NS1) belonging to the smallest segment (segment eight, NS) of the IAV using a reverse genetics system, and the GFP-encoding segment eight rescue plasmid was cotransfected into HEK293 cells with the remaining seven rescue plasmids to rescue the PR8-GFP virus. The rescued PR8-GFP virus was then amplified in the specific-pathogen-free embryonated chicken eggs.

### 2.2. Antibodies

The antibodies used in this study were as follows: antibodies for Flag (M2) (8146) were purchased from Cell Signaling Technology. Strep (2-1509-001) antibody was purchased from IBA Life Sciences. For the detection of Tom40 (sc-365467), α Tubulin (sc-8035), and β-actin (sc-47778) proteins and GST (sc-138), antibodies were purchased from Santa Cruz Biotechnology. The antibody for V5 (A190-220A) was purchased from BETHYL Laboratories. From Cell Signaling Technology, hMAVS (3993), pTBK1/NAK (D52C2; 5483), TBK-1 (D1B4; 3504), pIRF3 (4D4G; 4947), IRF3 (D83B9; 4302), pSTAT1 (58D6; 9167), STAT1 (42H3; 9175), pP65 (C22B4; 4764S), and P65 (S536, 93H1; 3033S) antibodies were purchased. The antibody for pMAVS (14341-1-AP) was purchased from Proteintech (Rosemont, IL, USA).

### 2.3. Plasmids

To construct the FMDV VP3 of the O1/Manisa/Turkey/69 strain, gene-specific PCR primers were used, and the PCR product was cloned into pIRES-Flag, pIRES-V5, pEXPR-Strep, and pEBG-GST expression vectors. Full-length MAVS was cloned into pIRES-Flag, pEBG-GST, pEXPR-Strep, expression vectors, and MAVS deletion mutants carrying each domain were cloned into pEBG expression vector tagged with GST.

### 2.4. Plasmid Transfection and Virus Infection

The plasmids were transfected to HEK293T cells with PEI reagent, and for all other cells, Lipofectamin 2000 (Invitrogen, Waltham, MA, USA) was used according to the manufacturer’s instructions. Before virus infection of the cells, the culture medium was changed with DMEM, containing 1% FBS and 1% antibiotic-antimycotic, and infected into target cells with the multiplicity of infection (MOI). Following 2 h of incubation at 37 °C, the extracellular virus was removed and replaced with 10% FBS containing DMEM.

### 2.5. Virus Titer Determination

Virus-infected cell lysate and culture supernatants were collected for the indicated times and virus titers were measured by plaque assay using Ceropithecus aethiops epithelial kidney (Vero) cells. The Vero cells were seeded into 12-well plates and, following 12 h of incubation at 37 °C, serially diluted virus-infected cell culture supernatant mixed with cell lysate was inoculated into Vero cells and incubated for 2 h at 37 °C with 1% DMEM. After 2 h incubation, the inoculum was removed and replaced with DMEM containing 0.1% agarose (Sigma-Aldrich, St. Louis, MO, USA). Then, cells were incubated for another 36 h at 37 °C and examined for plaque formation under 200× magnification. Virus titer was expressed as plaque-forming units per milliliter (PFU/mL) which was calculated using the number for plaque-forming units and the dilution factor.

### 2.6. Enzyme-Linked Immunosorbent Assay (ELISA)

ELISA was performed to detect the secreted IFNs and proinflammatory cytokines in cell culture supernatants. Mouse IL-6 (BD Biosciences, 555240), mouse interferon-β (CUSABIO, CSB-E04945m), human IL-6 (BD Biosciences, 555220), human interferon-β (CUSABIO, CSB-E09889h), porcine IL-6 (R&D Systems, P6000B), and porcine interferon-β (CUSABIO, CSB-E09890p) were used for the analysis according to the manufacturer’s protocols.

### 2.7. Quantitative Real-Time PCR

The RNeasy Mini Kit (Qiagen, Hiden, Germany) was used for the isolation of total RNA from the cells and cDNA was synthesized using reverse transcriptase (Toyobo, Osaka, Japan). QuantiTect SYBR Green PCR Kit (Toyobo) was used according to the manufacturer’s instructions with the primers listed in Table 1 for the qRT-PCR analysis on a Rotorgene instrument (Qiagen). The mRNA expression levels were analyzed according to the delta–delta CT (2^−ΔΔCT^) method, and β-actin or glyceraldehyde-3-phosphate dehydrogenase (GAPDH) was used as an internal housekeeping gene for normalization.

### 2.8. Luciferase Reporter Assay

HEK293T cells were transfected with IFN-β and thymidine kinase promoter-Renilla (TK-Renilla) luciferase reporter plasmids using PEI reagent. To stimulate IFN-β promoter luciferase, plasmids carrying the RIG-I, MDA5, MAVS, TRIF, TRAF3, or TBK1 gene were transfected together with luciferase reporter plasmids. At 24 h post-transfection, cells were lysed with 1× passive lysis buffer (Promega) and assayed for dual-luciferase activity using a dual-luciferase assay reagent kit (Promega; E1980) according to the manufacturer’s instructions. All the data are presented in accordance with relative firefly luciferase activity normalized against Renilla luciferase activities.

### 2.9. Immunoprecipitation Assay

Cells were harvested at 48 h post-transfection of target plasmids, and lysis with radioimmunoprecipitation assay (RIPA) lysis buffer (50 mM Tris-HCl, 150 mM NaCl, 0.5% sodium deoxycholate, 1% IGEPAL, 1 mM NaF, 1 mM Na_3_VO_4_) together with protease inhibitor cocktail and phosphatase inhibitor cocktail (Sigma) were used to obtain the whole-cell lysates (WCL). The WCL was precleared with Sepharose 6B (GE Healthcare Life Science, Chicago, IL, USA) at 4 °C for 2 h. The precleared WCL was used for immunoprecipitation. For GST or Strep pulldown, the WCL was incubated with a 50% slurry of glutathione-conjugated Sepharose (GST) beads or Strep-Tactin Sepharose Strep beads (IBA Solutions for Life Sciences, Göttingen, Germany), respectively, for 12 h at 4 °C. For Flag immunoprecipitation (IP), WCL was incubated with Flag antibody (1.0 µg/mL) for 12 h and then incubated with Protein A/G Plus agarose beads (Santa Cruz) for 4 h at 4 °C. The immunoprecipitated beads collected after centrifugation were washed with lysis buffer under different washing conditions and used for the immunoblot analysis.

### 2.10. Immunoblot Analysis

For immunoblot analysis, cell lysates or immunoprecipitated beads were mixed with 2× sample buffer (Sigma), and samples were loaded onto SDS-PAGE for the separation of the proteins according to their molecular weight. The proteins on SDS-PAGE gel were then transferred onto a PVDF membrane (Bio-Rad) using a Trans-Blot semi-dry transfer cell (Bio-Rad, Seoul, Korea) with buffer containing 30 mM Tris, 200 mM glycine, and 20% methanol. Following the transformation, the membrane was blocked with 5% bovine serum albumin for 1 h at room temperature and incubated with primary antibody at 4 °C overnight. Following that, the membrane was washed with either 1× PBST or TBST and incubated for 2 h with horseradish peroxidase-conjugated (HRP) secondary antibody at room temperature. Then, it was washed again with either 1× PBST or TBST and the membrane was developed with Western blotting detection reagents (ECL-GE Healthcare, Little Chalfont, UK) and visualized using a Las-4000 mini Lumino Image Analyzer.

### 2.11. Immunofluorescence and Confocal Microscopy

HEK293T cells were seeded into an eight-well chamber slide (ibidi). For each experiment, cells were fixed with 4% paraformaldehyde for 20 min at room temperature. After the fixation, cells were washed with 1× PBS and permeabilized by adding 100% methanol, followed by incubation for 20 min at −20 °C. Then, cells were again washed with 1× PBS and blocked with 2% BSA in 1× PBS for 1 h at room temperature, followed by incubation with relevant primary antibodies at 4 °C overnight. Next, cells were washed with 1× PBST three times and incubated with an appropriate secondary antibody for 1 h at room temperature. Then, cells were again washed three times with 1× PBST and stained with DAPI (4′,6-diamidino-2-phenylindole) for 10 min at room temperature. Images were taken under Nikon laser scanning confocal microscope (C2plus) and analyzed using NIS-Elements software.

### 2.12. MAVS Aggregation Assay

MAVS aggregation assay was performed according to the published protocol [18]. In brief, mitochondria were isolated from the cells using the Mitochondria isolation kit (Thermo 89874). A mitochondria pellet was suspended in 1× sample buffer (0.5× TBE, 10% glycerol, 2% SDS, and 0.0025% bromophenol blue) and subjected to semi-denaturing detergent agarose gel electrophoresis (SDD-AGE). For SDD-AGE, samples were loaded onto a 1.5% vertical agarose gel (1× TBE and 0.1% SDS) and electrophoresis in the running buffer (1× TBE and 0.1% SDS) for 50 min with a voltage of 100 V at 4 °C. Finally, the proteins were transferred to the immunoblot membrane (Millipore) for immunoblotting.

### 2.13. Statistical Analysis

Statistical analysis was performed using GraphPad Prism software version 6 for Windows. All the data are representative of at least three independent experiments and were presented as the means ± standard deviations (S.D.). An unpaired *t*-test was performed at each time point to compare the control and treatment groups. * *p* < 0.05 or ** *p* < 0.01 was regarded as significant.

## 3. Results

### 3.1. FMDV VP3 Negatively Regulate the Antiviral Responses

The FMDV VP3 structural protein plays a significant role in suppressing antiviral responses [46,47], but the underlying molecular mechanism remains unclear. To investigate how FMDV VP3 antagonizes antiviral responses, we used FMDV VP3 from the O1/Manisa/Turkey/69 strain.

**Figure 1:**
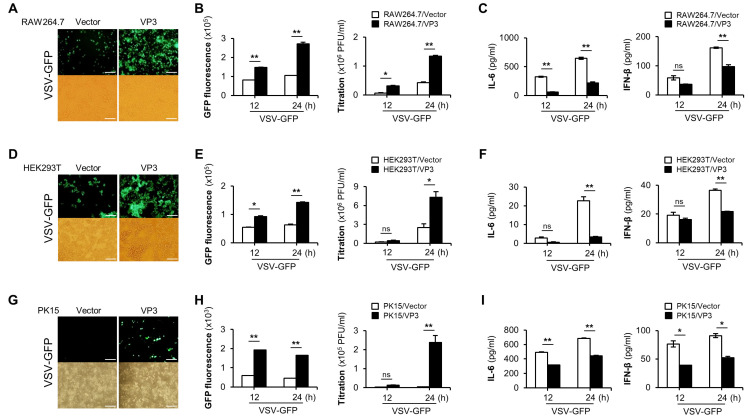
FMDV VP3 negatively regulates the antiviral responses. (**A**) GFP expression, (**B**) GFP fluorescence and virus titer, and (**C**) IL-6 and IFN-β secretion were measured in FMDV VP3 stably expressing or control Raw264.7 cells after VSV-GFP (1MOI) infection. Similarly, (**D**,**G**) GFP expression, (**E**,**H**) GFP fluorescence and virus titer, and (**F**,**I**) IL-6 and IFN-β secretion were measured in control plasmid or FMDV VP3 plasmid transiently transfected HEK293T and PK15 cells after VSVS-GFP (1MOI) infection. All the values are given as mean ± SD of two biological replicates. Scale bar represents 50 μM. Data are representative of three independent experiments, each with similar results. Student’s *t*-test; * *p* < 0.05; ** *p* < 0.01; ns, not significant.

To evaluate the role of FMDV VP3 in innate immune responses, we used VSV-GFP to infect FMDV VP3 stably expressing RAW264.7 cells (Figure 1A–C), HEK293T cells transiently transfected with FMDV VP3 (Figure 1D–F), and PK15 cells transiently transfected with FMDV VP3 (Figure 1G–I). By confirming the previous evidence, we observed higher VSV-GFP replication (Figure 1A,B) and lower levels of IL-6 and IFN-β production (Figure 1C) in FMDV VP3-overexpressing RAW264.7 cells than in control cells. Additionally, consistent with the results obtained in RAW264.7 cells, HEK293T and PK15 cells exhibited higher rates of virus replication (Figure 1D,E,G,H) and lower levels of IL-6 and IFN-β production (Figure 1F,I) when FMDV VP3 was overexpressed. Moreover, to determine whether the negative regulatory function mediated by FMDV VP3 is shared by different RNA virus-induced antiviral responses, we infected PR8-GFP virus into PK15 cells transiently transfected with a FMDV VP3 expression plasmid or a control plasmid (Figure S1). Consistent with the results obtained with VSV-GFP infection, we observed higher levels of PR8-GFP replication and lower levels of IL-6 and IFN-β secretion in FMDV VP3-expressing PK15 cells (Figure S1). In addition, we further evaluated the IFN-suppressive function of FMDV VP3 belonging to seven FMDV serotypes, and in the results, they showed virus replication phenotypes similar to the FMDV VP3 of O1/Manisa/Turkey/69 (Figure S2).

Taken together, these results suggest that, irrespective of the cell type, FMDV VP3 negatively regulates antiviral responses, and that the impact of FMDV VP3 on RNA virus-induced antiviral responses is not specific to the virus. Moreover, the IFN-suppressive function of FMDV VP3 is conserved among all FMDV serotypes.

### 3.2. FMDV VP3 Suppress Virus-Mediated Type-I IFN Signaling and Antiviral Gene Transcription

**Figure 2:**
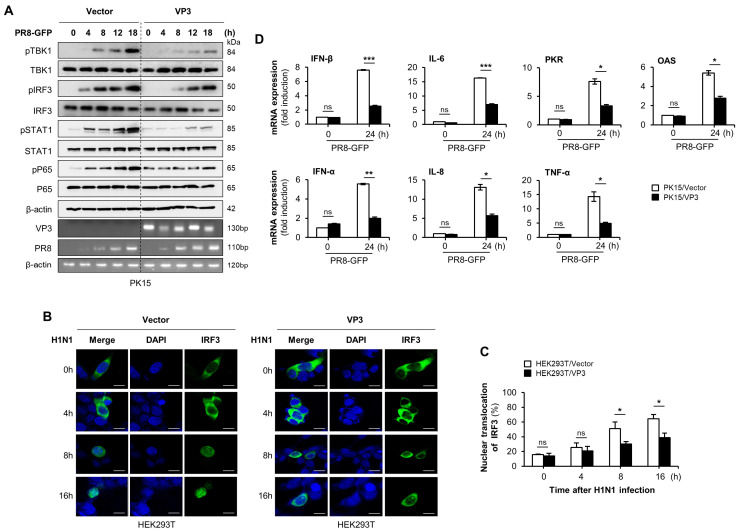
FMDV VP3 inhibits the activation of key molecules in the type-I IFN signaling cascade and suppresses antiviral gene expression. (**A**) Control plasmid or FMDV VP3 plasmid transiently transfected PK15 cells were infected with PR8-GFP (3MOI). At the indicated times after infection, TBK1, IRF3, STAT1, P65, and phosphorylated TBK1, IRF3, STAT1, and P65 protein levels in PK15 cell extracts were detected by immunoblotting, followed by the qRT-PCR for FMDV VP3, PR8-GFP virus NS1 protein, and β-actin. The β-actin was used to confirm the equal loading of proteins for immunoblotting. (**B**) HEK293T cells were transfected with control or FMDV VP3 plasmid together with GFP-IRF3 (green) plasmid, followed by infection with H1N1 influenza virus (1MOI). Cells were fixed at indicated time points and DAPI (blue) was used to stain the nuclei. Scale bar represents 5 μM. (**C**) The percentages of cells showing nuclear translocation of IRF3 were calculated by dividing the number of cells exhibiting nuclear expression of IRF3 by the total number of GFP-positive cells. (**D**) Control plasmid or FMDV VP3 plasmid transiently transfected PK15 cells were infected with PR8-GFP (3MOI). At indicated time points after infection, total RNAs were extracted from the cells, and expression of the indicated mRNA-encoding antiviral genes was analyzed by qRT-PCR. All the data are representative of at least two independent experiments, each with similar results, and the values are expressed as mean ± SD of two biological replicates. Student’s *t*-test; * *p* < 0.05; ** *p* < 0.01; *** *p* < 0.001; ns, not significant.

To further investigate the effect of FMDV VP3 on the virus-mediated type-I IFN signaling cascade, we monitored the virus-induced phosphorylation of TBK1, IRF3, STAT1, and p65. To this end, PK15 cells transiently transfected with FMDV VP3 or control plasmid were stimulated with PR8-GFP, and samples were harvested at the indicated time points. Cell lysates were analyzed by immunoblotting to detect the phosphorylation levels of molecules involved in type-I IFN signaling. Phosphorylation levels of these proteins were lower in cells expressing FMDV VP3, and these differences grew over time (Figure 2A). We observed a similar pattern of phosphorylation levels in FMDV VP3 stably expressing or control RAW264.7 cells (Figure S3). To further validate the inhibitory effect of FMDV VP3 on type-I IFN signaling, we examined IRF3 localization in FMDV VP3-overexpressing and control HEK293T cells following viral infection. After infection of control HEK293T cells with H1N1, the percentage of nuclear-translocated IRF3 increased to 65% at 16 h post-infection (hpi). By contrast, the percentage of nuclear-localized IRF3 was lower in FMDV VP3-overexpressing HEK293T cells: ~40% at 16 hpi (Figure 2B,C).

In addition, we examined the mRNA expression of IFN-β and IFN-related antiviral genes (IL-6, PKR, and OAS) to determine whether the inhibitory effect of FMDV VP3 on type-I IFN signaling influences the transcription levels of those genes. For these experiments, mRNA was isolated from FMDV VP3 or control plasmid transiently transfected PK15 cells at the indicated time points, following PR8-GFP infection. Consistent with the phosphorylation levels of type-I IFN signaling molecules, the levels of mRNA encoding IFN and IFN-related antiviral genes were lower in PK15 cells expressing FMDV VP3 (Figure 2D). Similar patterns of mRNA expression were observed in both RAW264.7 and HEK293T cells (Figure S3). Collectively, these results indicate that the reduced antiviral response was related to FMDV VP3-mediated negative regulation of the type-I IFN signaling cascade and the resultant suppression of antiviral gene expression.

### 3.3. FMDV VP3 Predominantly Targets the TM Domain of MAVS to Antagonize IFN-β Production

**Figure 3:**
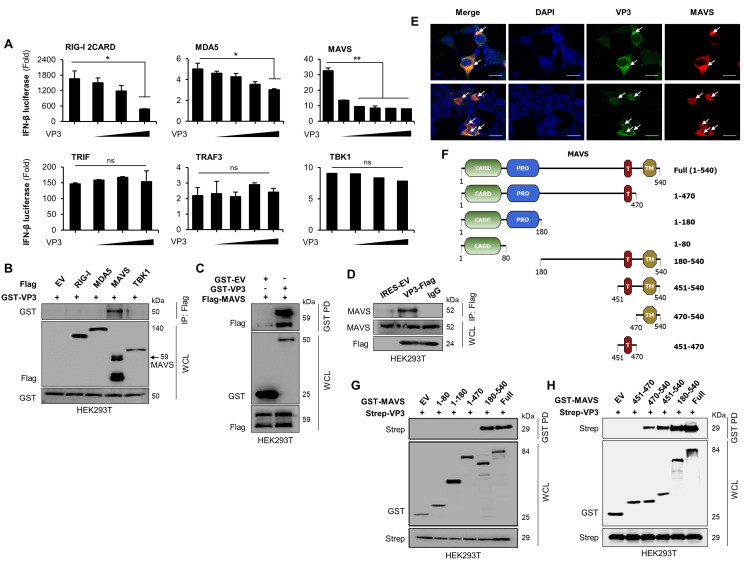
FMDV VP3 binds to the transmembrane domain of MAVS. (**A**) HEK293T cells were transfected with an IFN-β reporter plasmid, plus TK-Renilla plasmid and increasing amounts of FMDV VP3 plasmid, along with overexpression plasmids for RIG-I, MDA5, MAVS, TRIF, TRAF3, or TBK1. At 24 h post-transfection, luciferase activity was measured in a luminometer. TK-Renilla was used as transfection control to normalize firefly luciferase activity. (**B**) HEK293T cells were cotransfected with the control vector (Flag), Flag-tagged RIG-I, MDA5, MAVS, and TBK1 plasmids together with GST-tagged FMDV VP3 plasmids. Cell lysates were subjected to immunoprecipitation (IP), followed by immunoblotting with an anti-GST antibody. WCL was immunoblotted with anti-Flag and anti-GST antibodies. (**C**) HEK293T cells were cotransfected with the control vector (GST), Flag-MAVS, and GST-tagged FMDV VP3 plasmid. Cell lysates were subjected to GST pulldown (PD), followed by immunoblotting with an anti-Flag antibody. Whole-cell lysate (WCL) was immunoblotted with anti-GST and anti-Flag antibodies. Lysates of (**D**) HEK293T cells transfected with control vector (Flag) or with Flag-tagged FMDV VP3 plasmid were subjected to immunoprecipitation with Flag antibody or control IgG, followed by immunoblotting with anti-MAVS antibody. WCL was immunoblotted with anti-MAVS and anti-Flag antibodies. (**E**) HEK293T cells were transfected with Flag-tagged MAVS plasmid together with V5-tagged FMDV VP3 plasmid, followed by confocal microscopy assay with anti-Flag (red) and anti-V5 (green) antibodies. Nuclei were stained with DAPI (blue). Scale bar represents 5 μM. Arrow indicates the co-localized VP3 and MAVS protein. (**F**) GST-tagged full-length and deletion mutants of MAVS were constructed for the immunoprecipitation assay. (**G**) GST-tagged full length and amino acid 1–80, 1–180, 1–470, and 180–540 fragments of MAVS, and (**H**) GST-tagged full length and amino acid 451–470, 470–540, 451–540, and 180–540 fragments of MAVS or control vector (GST) were cotransfected to HEK293T cells together with Strep-tagged FMDV VP3 plasmid. Cell lysates were subjected to GST-PD and immunoblotted with anti-Strep antibody following immunoblotting of the WCL with both anti-Strep and anti-GST antibodies. In A, data are representative of three independent experiments, each with similar results, and all the values are expressed as mean ± SD of two biological replicates. All the immunoblot and confocal data are representative of at least two independent experiments, each with similar results. Student’s *t*-test; * *p* < 0.05; ** *p* < 0.01; ns, not significant.

Host sensor molecules detect viral infections and activate the type-I IFN signaling cascade to induce antiviral responses. The observations described above and in previous studies [44] show that FMDV VP3 negatively regulates this signaling pathway. Hence, to determine how FMDV VP3 affects the type-I IFN cascade, we performed IFN-β luciferase promoter assays in HEK293T cells. In these experiments, the cells co-expressed FMDV VP3 along with several molecules involved in the type-I IFN cascade. Luciferase assays revealed that FMDV VP3 inhibited RIG-I-, MDA5-, and MAVS-mediated IFN-β promoter activity in a dose-dependent manner (Figure 3A). However, we observed no detectable change in TRIF-, TRAF3-, and TBK1-mediated IFN-β promoter activity as a function of FMDV VP3 dose (Figure 3A). Because TRIF is an adapter molecule for the TLR3 and connects to the RIG-I–mediated type-I IFN cascade at TRAF3, and TBK1 acts downstream of TRAF3, the lack of a detectable change in TRAF3-mediated IFN-β promoter activity in the presence of FMDV VP3 suggests that FMDV VP3 targets a molecule immediately upstream of TRAF3 in the RIG-I–mediated type-I IFN pathway. Because MAVS is immediately upstream of TRAF3, we hypothesized that FMDV VP3 targets the MAVS signaling complex to negatively regulate the type-I IFN responses. Hence, to confirm the selectivity of the FMDV VP3–MAVS interaction among the molecules of the type-I IFN pathway, we performed immunoprecipitation assays with RIG-I, MDA5, MAVS, and TBK1. The results clearly demonstrated the selectivity of the FMDV VP3–MAVS interaction (Figure 3B). To further confirm the relationship between FMDV VP3 and MAVS, we examined their physical interaction by immunoprecipitation assay in HEK293T cells co-transfected with GST-tagged FMDV VP3 and MAVS-FLAG. The results of co-immunoprecipitation revealed a clear association between MAVS and FMDV VP3 (Figure 3C). We observed an interaction between FMDV VP3 and endogenous MAVS in HEK293T cells (Figure 3D). In addition, we confirmed the colocalization of FMDV VP3 with MAVS by confocal microscopy (Figure 3E).

Previous studies have suggested that FMDV VP3 interacts with the TM domain of MAVS [46], but the downstream mechanism of type-I IFN signaling inhibition remains unclear. Hence, to confirm the previous findings, we constructed a series of GST-tagged MAVS deletion mutants (Figure 3F) and performed co-immunoprecipitation assays with Strep-tagged FMDV VP3. For these experiments, full-length and truncated forms (aa 1–80, 1–180, 1–470, and 180–540) of MAVS were transiently transfected into HEK293T cells along with Strep-tagged FMDV VP3. The results of co-immunoprecipitation revealed a clear association of FMDV VP3 with the full-length and C-terminal region of MAVS (aa 180–540), whereas C-terminal truncations of MAVS (aa 1–80, 1–180, and 1–470) lost the ability to interact with FMDV VP3 (Figure 3G). Next, to identify the specific MAVS motif that binds to FMDV VP3, we co-transfected GST-tagged full-length MAVS or N-terminally truncated deletion mutants along with Strep-tagged FMDV VP3 into HEK293T cells, and then performed co-immunoprecipitation assays. Full-length and N-terminally truncated MAVS deletion mutants (aa 470–540, 451–540, and 180–540), all of which contain the transmembrane (TM) domain, clearly interacted with FMD VP3, whereas amino acids (aa) 451–470 of MAVS did not (Figure 3H). Taken together, these findings indicate that FMDV VP3 predominantly targets the TM domain of MAVS (aa 470–540) to negatively regulate type-I IFN responses.

### 3.4. FMDV VP3 Inhibits Mitochondrial Localization, Self-Association, and Aggregation of MAVS

**Figure 4:**
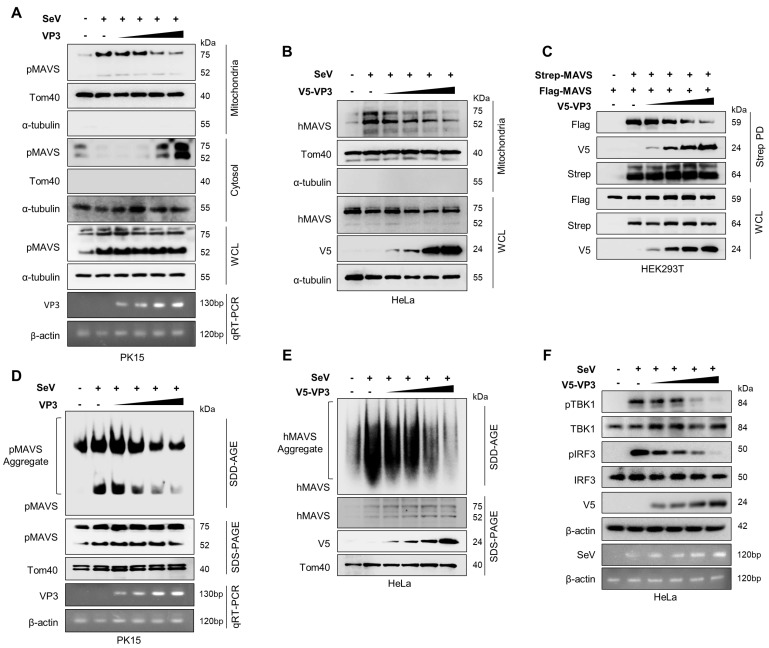
FMDV VP3 disrupts MAVS activation. (**A**,**B**) Immunoblot analysis of mitochondria fraction isolated from the (**A**) PK15 and (**B**) HeLa cells which transfected with increasing amounts of V5-tagged FMDV VP3 expression plasmid, and Sendai virus (SeV) infected (1MOI). Mitochondria fraction was immunoblotted with the MAVS, Tom40, and α-tubulin antibodies. PK15 cell whole-cell lysate (WCL) was immunoblotted with MAVS and α-tubulin antibodies, while HeLa cell WCL was immunoblotted with anti-MAVS, anti-V5, and anti-α-tubulin antibodies. In PK15 cells, qRT-PCR was done to detect the expression of FMDV VP3. (**C**) HEK293T cells were cotransfected with control vector (Strep), Flag-MAVS, Strep-MAVS, and increasing doses of V5-tagged FMDV VP3 plasmid. The cell lysates were subjected to Strep-PD and subsequent immunoblotting with anti-Flag, anti-V5, and anti-Strep antibodies. Further, WCL was immunoblotted with anti-Flag, anti-Strep, and anti-V5 antibodies. (**D**) PK15 and (**E**) HeLa cells were transfected with increasing amounts of V5-tagged FMDV VP3 plasmid and infect the SeV (1MOI). Following that, crude mitochondria were isolated from the cells and subjected to the semi-denaturing detergent agarose gel electrophoresis (SDD-AGE) and immunoblotted with anti-MAVS antibody for the MAVS aggregation detection. The same sample was used for the SDS-PAGE and immunoblotted with anti-MAVS and anti-Tom40 antibodies for PK15 cells, and anti-MAVS, anti-V5, and anti-Tom40 antibodies for HeLa cells. In PK15 cells, qRT-PCR was done to detect the expression of FMDV VP3. (**F**) HeLa cells were transiently transfected with the increasing amounts of V5-tagged FMDV VP3 plasmid and infected with SeV (1MOI). Cell lysates were immunoblotted against phosphorylated (p-) TBK1, TBK1, pIRF3, IRF3, V5, and β-actin antibodies, followed by the qRT-PCR for SeV C protein, and β-actin. All the data are representative of two independent experiments, each with similar results.

Previous studies demonstrated that the TM domain of MAVS is crucial for the mitochondrial localization of MAVS, and its subsequent self-association and aggregation, which result in IFN production to promote viral clearance [20,21,22,48,49,50]. Our results suggest that FMDV VP3 specifically targets the TM domain of MAVS to disrupt the mitochondrial localization of MAVS, thereby preventing its self-association and aggregation, to antagonize IFN signaling. To explore this possibility, we investigated the impact of FMDV VP3 on mitochondrial localization of MAVS in PK15 cells. For these experiments, PK15 cells were transiently transfected with the indicated amounts of V5-tagged FMDV VP3 plasmid, and then infected with Sendai Virus (SeV) to activate the type-I IFN pathway. At 24 hpi, the mitochondrial fractions of the cells were isolated and immunoblotted to detect mitochondria-localized MAVS. Fractionation revealed a marked inhibition of MAVS mitochondrial localization in the presence of increasing amounts of FMDV VP3 (Figure 4A). Similar results were obtained in HeLa cells (Figure 4B). Next, we evaluated the effect of FMDV VP3 on MAVS self-association by transfecting HEK293T cells with Strep-tagged and FLAG-tagged MAVS expression plasmids, along with increasing amounts of V5-tagged FMDV VP3 plasmids. Strep-tagged MAVS was immunoprecipitated using Strep-Tactin Sepharose Strep beads, and FLAG-tagged MAVS and V5-tagged FMDV VP3 were detected using anti-FLAG and anti-V5 antibodies, respectively. Coimmunoprecipitation assays demonstrated that MAVS self-association decreased dramatically as the level of FMDV VP3 increased (Figure 4C). We then examined the impact of FMDV VP3 on MAVS aggregation in PK15 cells. For this purpose, PK15 cells were transiently transfected with the indicated amounts of V5-tagged FMDV VP3 plasmids and then infected with SeV to induce the type-I IFN signaling cascade. At 24 hpi, we isolated total mitochondria from the cells and subjected them to semi-denaturing detergent agarose gel electrophoresis (SDD-AGE) to detect MAVS aggregation. The results of this analysis revealed clear inhibition of MAVS aggregation as the level of FMDV VP3 increased in PK15 cells (Figure 4D). Similar results were obtained in HeLa cells (Figure 4E). Finally, we examined the impairment of the downstream type-I IFN signaling cascade due to the FMDV VP3-mediated inactivation of MAVS by evaluating the phosphorylation levels of TBK1 and IRF3, which are downstream of MAVS in the type-I IFN pathway. We transiently transfected HeLa cells with different amounts of V5-tagged FMDV VP3 expression plasmid, induced with SeV, and then detected the levels of virus-induced phosphorylation of TBK1 and IRF3. The phosphorylation level reduction upon the increased expression levels of FMDV VP3 shows a clear inactivation of the type-I IFN signaling cascade downstream to MAVS (Figure 4F).

Collectively, these data suggest that FMDV VP3 specifically targets the TM domain of MAVS to obstruct its mitochondrial localization, thereby impairing MAVS self-association, aggregation, and downstream activation of IFN signaling.

## 4. Discussion

RLR- and TLR-mediated type-I IFN production is an extremely powerful mechanism of host defense against invading viruses, including picornaviruses [9,11]. Accordingly, viruses employ a broad range of strategies to inhibit type-I IFN signaling, enabling them to replicate efficiently in the host [51,52,53]. To develop effective prevention strategies against emerging viruses, it is essential to characterize in precise detail the mechanisms of immune evasion used by pathogens [47,54].

FMDV has adopted multiple strategies to evade host type-I IFN responses [23,24]. For example, the structural protein VP3, a major capsid protein of FMDV, plays an important role in immune suppression by targeting the IFN signaling cascade [46,47]. Specifically, FMDV VP3 degrades Janus kinase 1 (JAK1) and thus inhibits IFN-ɣ signal transduction [47]. Importantly, FMDV VP3 inhibits transcription of MAVS mRNA, further contributing to type-I IFN suppression [46]. Since transcription is an upstream process to the protein translation, protein expression tends to reduce upon inhibition of mRNA transcription [55]. However, during our study, we did not observe a reduction in MAVS protein expression upon overexpression of FMDV VP3 (Figure 4C). The absence of a link between the FMDV VP3–MAVS interaction and transcriptional inhibition of MAVS means that the underlying mechanism remains unclear.

In this study, we aimed to elucidate the exact molecular mechanism of FMDV VP3-mediated suppression of type-I IFN signaling. First, we showed that the overexpression of FMDV VP3 negatively regulates the RNA virus-induced type-I IFN signaling cascade and subsequent IFN production, thereby increasing viral replication. Second, based on the results of the IFN-β luciferase reporter assay and immunoprecipitation assay, we confirmed that FMDV VP3 interacts with MAVS, and demonstrated that FMDV VP3 specifically targets the TM domain of MAVS (aa 470–540) to counteract IFN production. Third, we showed that FMDV VP3 inhibits the mitochondrial localization, self-association, and aggregation of MAVS. Taken together, these findings revealed that FMDV VP3 negatively regulates the type-I IFN signaling pathway by targeting MAVS and disrupting its activation.

Since TM domain of MAVS is positioned at mitochondrial membranes [21], and to a lesser extent, at peroxisomal membranes [18], we hypothesize that FMDV VP3 protein, which is localized in the cytoplasm, interacts with the newly synthesized MAVS before their membrane localization. This is because, upon the virus infection, expression of the type-I IFN pathway-related molecules [56,57], including MAVS, tends to increase due to the activation of the transcription and translation machinery [58,59] of those genes in the cytoplasm. For the mitochondrial localization of the newly synthesized protein in the cytoplasm, Tom70 protein on the outer mitochondrial membrane recognizes the newly synthesized mitochondrial proteins in the cytosol and plays a role in translocating them to their final destination in the mitochondria [60].

MAVS is predominantly localized to the mitochondrial membrane via its conserved C terminus hydrophobic TM domain [21,22]. Mitochondrial localization is important for the signaling function of MAVS [21,22,61], and the mitochondrial membrane provides a strategic position for sensing viral replication. Hence, MAVS cellular mislocalization significantly reduced its activity [21]. Upon viral infection, RIG-I detects viral RNA and catalyzes the self-association of MAVS on the mitochondrial membrane and subsequent conversion into very large prion-like aggregates that potently activate IRF3 [20,22,62,63]. These prion-based conformational switches serve as a mechanism that regulates protein functions and cellular phenotypes. Thus, the formation of MAVS aggregates plays a key role in the propagation of the antiviral signaling cascade and is tightly regulated by viral infection [20].

Because MAVS is a critical component of the type-I IFN antiviral signaling cascade [64], many viruses have evolved strategies for evading host antiviral immunity by targeting MAVS to achieve successful infection [65]. For example, the NS3-4A protease of the hepatitis C virus (HCV) specifically targets the C-terminal region of MAVS and cleaves at amino acid 508, resulting in the removal of the TM domain from MAVS [48]. This facilitates the subsequent dissociation of the MAVS molecular complex from the mitochondrial outer membrane and disrupts MAVS oligomerization, thereby inhibiting the antiviral immune response [48]. As with HCV, GB Virus B (GBV-B) uses the same strategy to disrupt RIG-I signaling via NS3/4A-mediated cleavage of MAVS, resulting in mislocalization of MAVS to the cytoplasm [66]. In addition to viral proteins, host factors also target MAVS to inhibit persistent antiviral signaling that would lead to immunopathology. For example, the mitochondrial-resident E3 ligase MARCH5 negatively regulates MAVS aggregation via proteasomal degradation [67]. Importantly, some energy metabolites also regulate type-I IFN production to connect energy metabolism and innate immunity [50]. Lactate, a key metabolite in the glycolytic pathway, inhibits the type-I IFN antiviral signaling by directly binding to the TM domain of MAVS, thereby preventing mitochondrial localization and subsequent aggregation of MAVS [50]. This is analogous to the mechanism that we identified in this study, in which FMDV VP3 negatively regulates type-I IFN production.

In summary, our results demonstrate that FMDV VP3 facilitates virus replication in the host cell by negatively regulating the type-I IFN antiviral signaling cascade. To inhibit type-I IFN production, FMDV VP3 specifically binds the TM domain (aa 470–540) of MAVS and disrupts its mitochondria localization, self-association, and aggregation, which are key events in the activation of downstream molecules in the type-I IFN antiviral signaling cascade. These findings reveal the exact molecular mechanism used by the FMDV VP3 to counteract the type-I IFN response and expand our knowledge about the immune evasion strategies used by FMDV to evade host immunity.

## Figures and Tables

**Table 1:** Primers for qRT-PCR.

Gene ^a^	Forward	Reverse
pIFN-β	AAATCGCTCTCCTGATGTGT	TGCTCCTTTGTTGGTATCG
pIL-6	CACCGGTCTTGTGGAGTTTC	GTGGTGGCTTTGTCTGGATT
pPKR	GAGAAGGTAGAGCGTGAAG	CCAGCAACCGTAGTAGAG
pOAS	CTGTCGTTGGACGATGTATGCT	CAGCCGGGTCCAGAATCA
pIFN-α	GCCTCCTGCACCAGTTCTACA	TGCATGACACAGGCTTCCA
pIL-8	TTTCTGCAGCTCTCTGTGAGG	CTGCTGTTGTTGTTGCTTCTC
pTNF-α	CCACGTTGTAGCCAATGTC	CTGGGAGTAGATGAGGTACAG
pβ-actin	CTCGATCATGAAGTGCGACG	GTGATCTCCTTCTGCATCCTGT
mIFN-β	TCCAAGAAAGGACGAACATTCG	TGCGGACATCTCCCACGTCAA
mISG-15	CAATGGCCTGGGACCTAAA	CTTCTTCAGTTCTGACACCGTCA
mISG-56	AGAGAACAGCTACCACCTTT	TGGACCTGCTCTGAGATTCT
mPKR	GCCAGATGCACGGAGTAGCC	GAAAACTTGGCCAAATCCACC
mOAS	GAGGCGGTTGGCTGAAGAGG	GAGGAAGGCTGGCTGTGATTGG
mGAPDH	TGACCACAGTCCATGCCATC	GACGGACACATTGGGGGTAG
hIFN-β	CATCAACTATAAGCAGCTCCA	TTCAAGTGGAGAGCAGTTGAG
hIL-6	CCACACAGACAGCCACTCACC	CTACATTTGCCGAAGAGCCCTC
hISG-15	GAGAGGCAGCGAACTCATCT	CTTCAGCTCTGACACCGACA
hISG-56	AAGGCAGGCTGTCCGCTTA	TCCTGTCCTTCATCCTGAAGCT
hTNF-α	ATGAGCACTGAAAGCAT	TCGACGGGGAGTCGAACT
hβ-actin	CCAACCGCGAGAAGATGACC	GATCTTCATGAGGTAGTCAGT
PR8-NS1	CAAACGAGTTGCAGACCAAG	TCTTGATGTCCAGACCGAGA
SeV-C	GGAGGAAGAGAGTCGCTCTC	TCCTTGGGGAGTGTTGATGG
FMDV-VP3	TAAGACCTCGGACCCCGTTTACG	ATACGGTACCCCGTTGAAATCGA

^a^ p, porcine; m, mouse; h, human.

## Data Availability

All relevant data are within the manuscript and its Supporting Information files.

## Supplementary Materials

The following are available online at https://www.mdpi.com/article/10.3390/v13091776/s1, Figure S1: FMDV-VP3 negatively regulates cellular antiviral response, Figure S2: Negative regulation of antiviral responses is conserved among the VP3 of seven FMDV serotypes, Figure S3: FMDV VP3 antagonist the type-I IFN pathway signaling cascade activation and antiviral gene expression.
