# Supplementary material for: Inhibition of MAVS Aggregation-Mediated Type-I Interferon Signaling by Foot-and-Mouth Disease Virus VP3
Source: Viruses. 2021 Sep 6;13(9):1776. doi: 10.3390/v13091776 (PMC8473216; doi:10.3390/v13091776)
Supplement: Supplementary file 1 [file viruses-13-01776-s001.zip › viruses-1334764-supplementary.pdf]

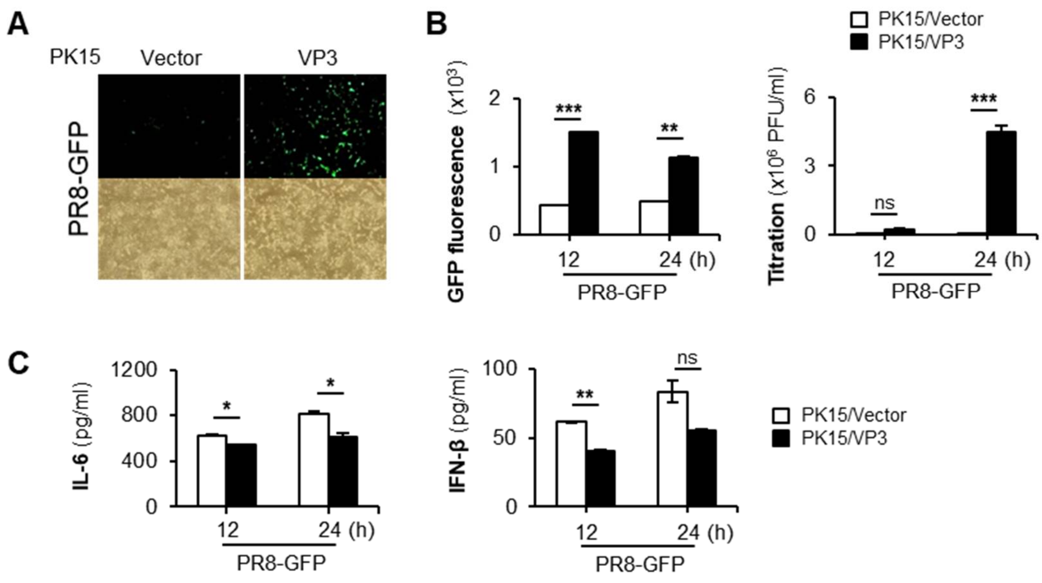

Figure S1. FMDV-VP3 negatively regulates cellular antiviral response. (A to C) PK15 cells were transiently transfected with a control plasmid or FMDV-VP3 expression plasmid and infected with PR8-GFP (3MOI). (A) GFP expression, (B) GFP fluorescence and virus titer, and (C) IL-6 and IFN-β secretion were measured at indicated time points. Data are representative of three independent experiments, each with similar results. Error bars indicate the mean ± SD of two biological replicates. Student's t-test; *$p < 0.05$; **$p < 0.01$; ***$p < 0.001$; ns, not significant.

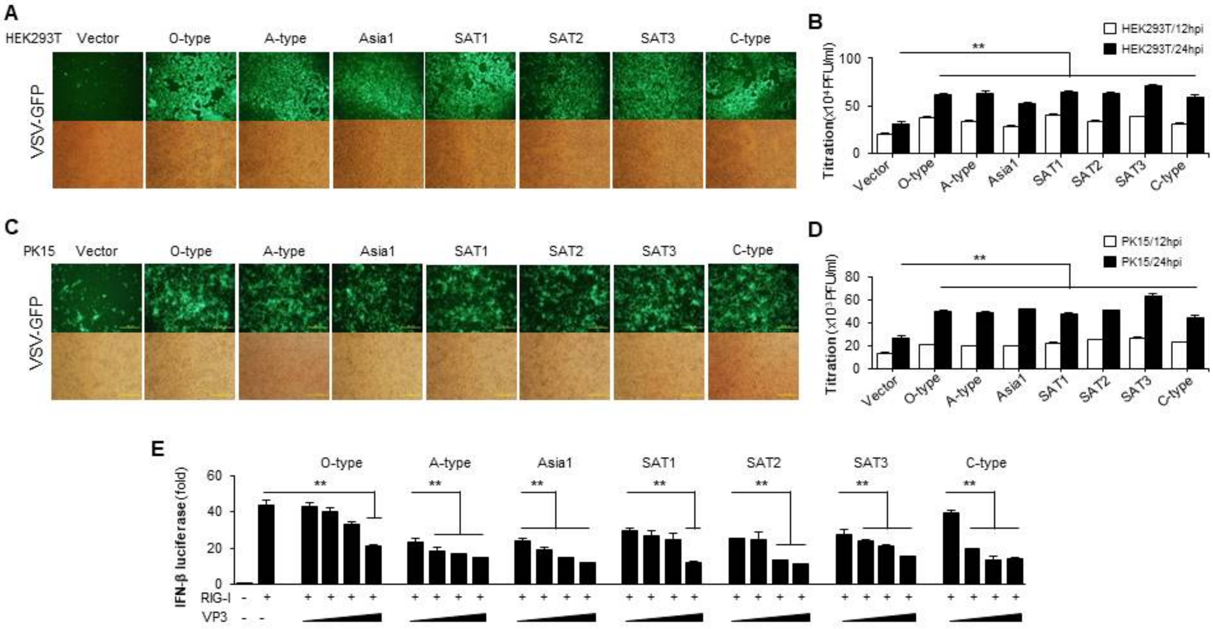

Figure S2. Negative regulation of antiviral responses is conserved among the VP3 of seven FMDV serotypes. (A and C) GFP expression and (B and D) virus titer was analyzed in control plasmid or individual plasmids that contain the VP3 of seven FMDV serotypes transfected HEK293T and PK15 cells after VSV-GFP (1MOI) infection. (E) IFN-β luciferase promoter assay was conducted in HEK293T cells transfected with firefly luciferase reporter plasmid encoding the IFN-β promoter, plus TK-Renilla plasmid and an increasing dose of Flag-tagged VP3 plasmids of seven FMDV serotypes, plus expression plasmids for RIG-I. At 24 h post transfection, luciferase activity was measured in a luminometer. Data are representative of at least three independent experiments, each with similar results. All the values are expressed as mean ± SD of two biological replicates. Student's t-test; $**p < 0.01$.

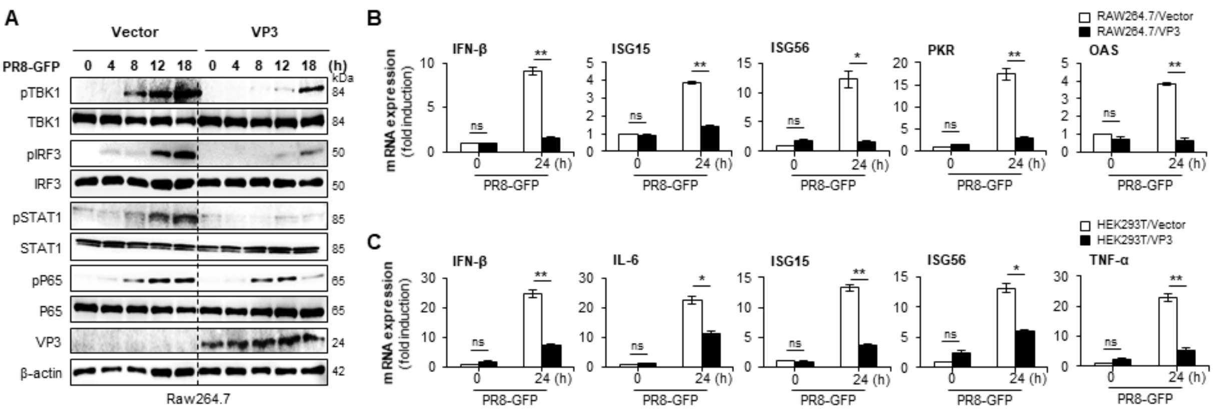

Figure S3. FMDV VP3 antagonist the type-I IFN pathway signaling cascade activation and antiviral gene expression. (A) FMDV VP3 stably expressing or control Raw264.7 cells were infected with PR8-GFP (3MOI). At the indicated times after infection, TBK1, IRF3, STAT1, P65, and phosphorylated TBK1, IRF3, STAT1, P65 protein levels in Raw264.7 cell extracts were detected by immunoblotting. The β-actin was used to confirm the equal loading of proteins for immunoblotting. (B) FMDV VP3 stably expressing or control Raw264.7 cells, (C) control plasmid or FMDV VP3 plasmid transiently transfected HEK293T cells were infected with PR8-GFP (3MOI). At indicated time points after infection, total RNAs were extracted from the cells, and expression of the indicated mRNA-encoding antiviral genes was analyzed by real-time quantitative reverse transcription-PCR (qRT-PCR). All the data are representative of at least two independent experiments, each with similar results, and the values are expressed as mean ± SD of two biological replicates. Student's t-test; *$p < 0.05$; **$p < 0.0$; ns, not significant.
